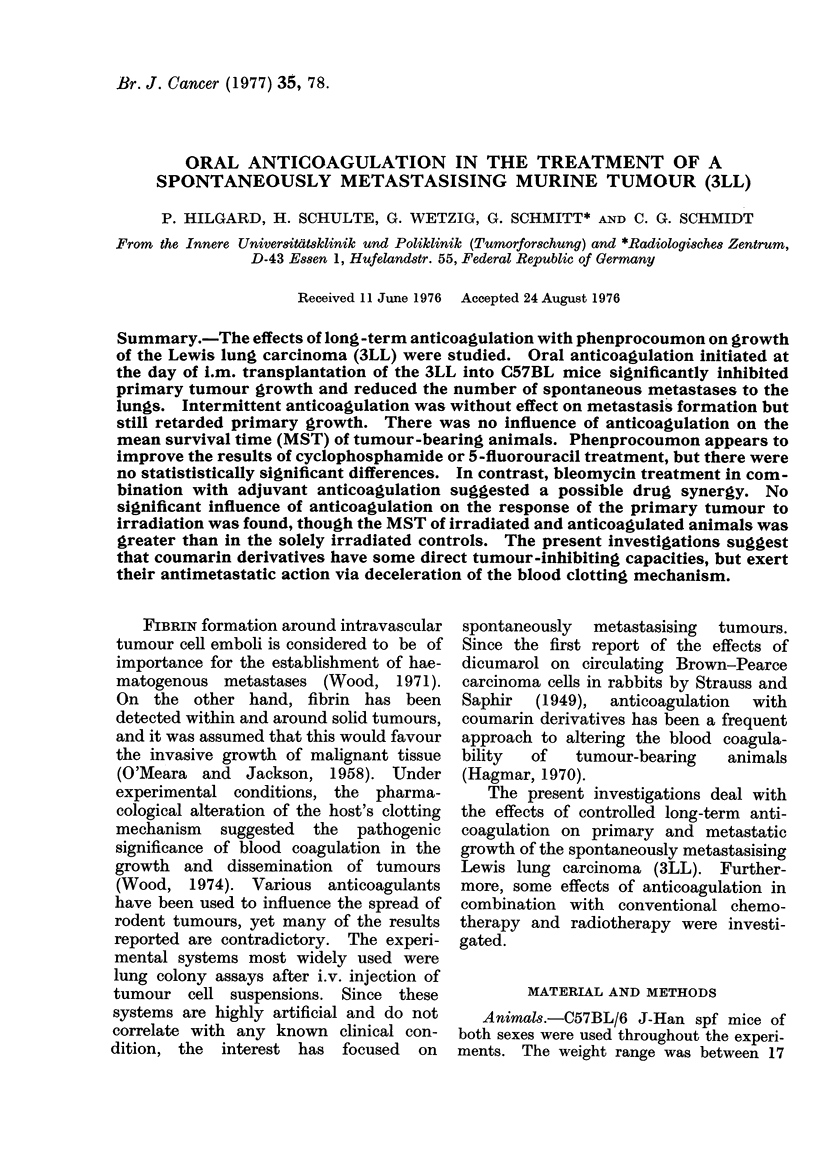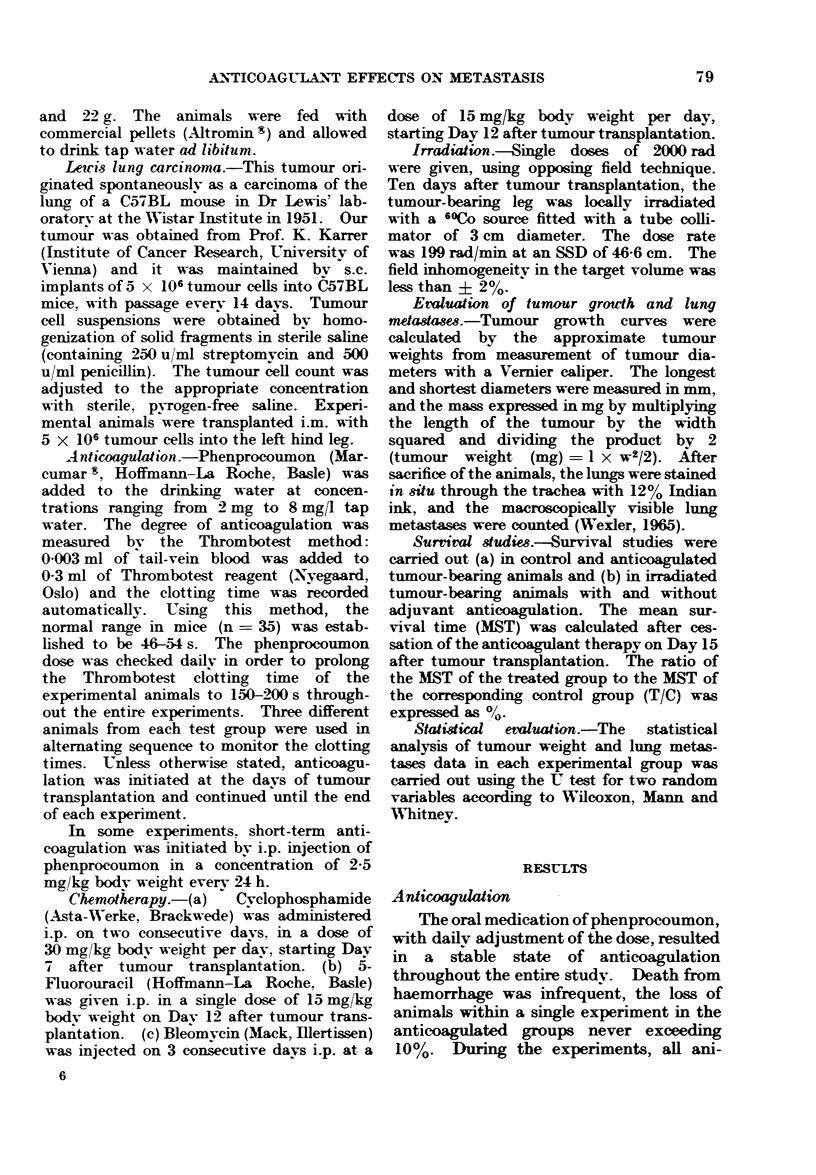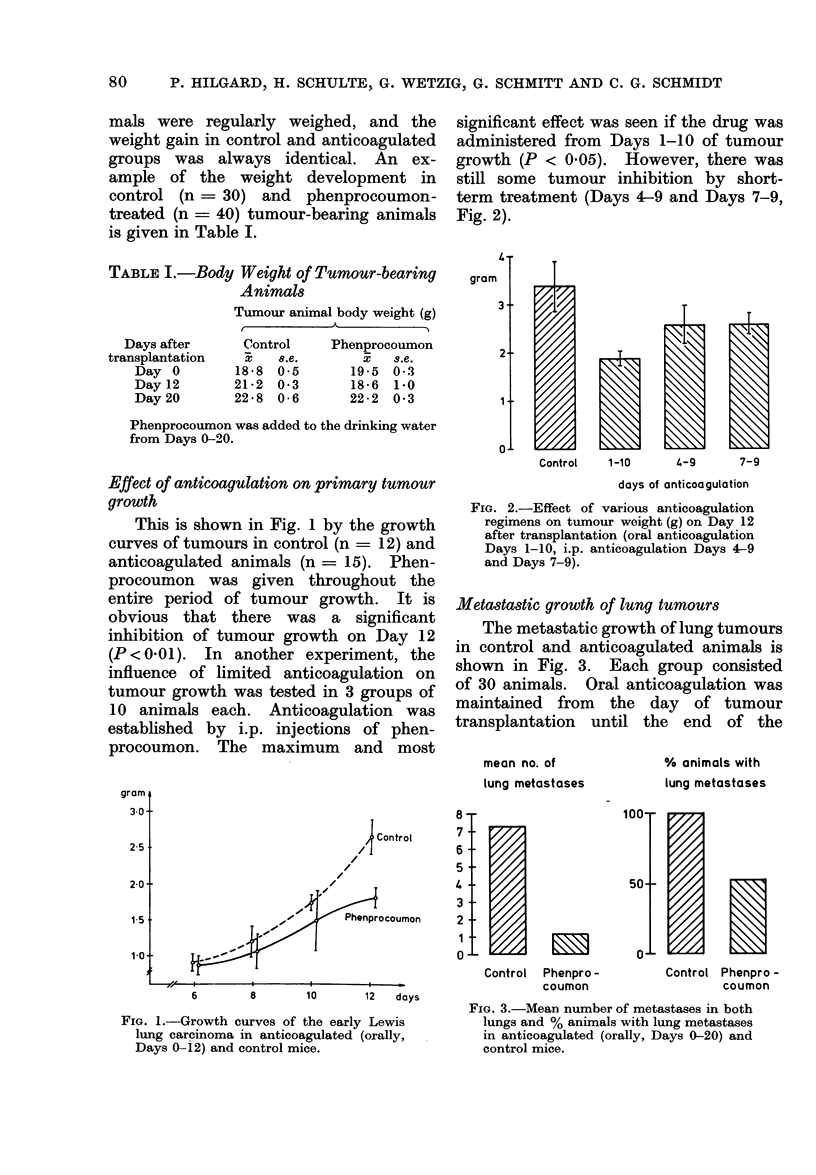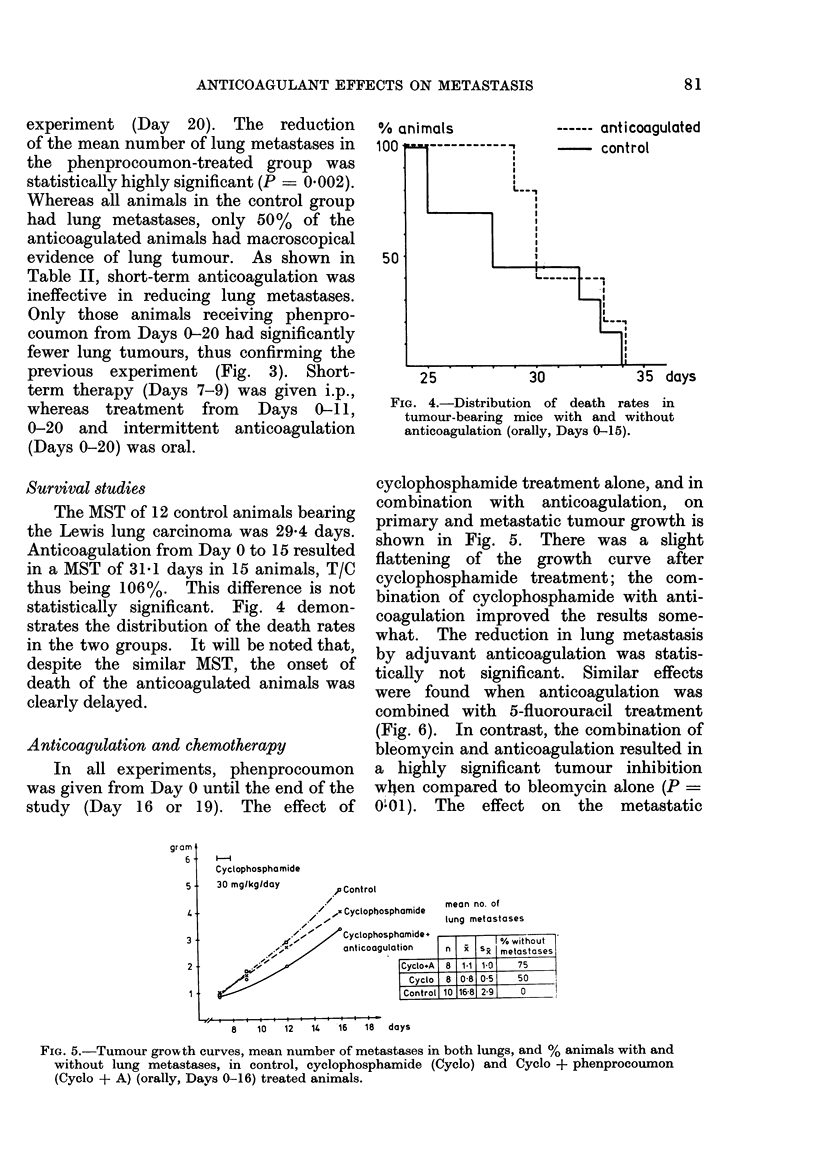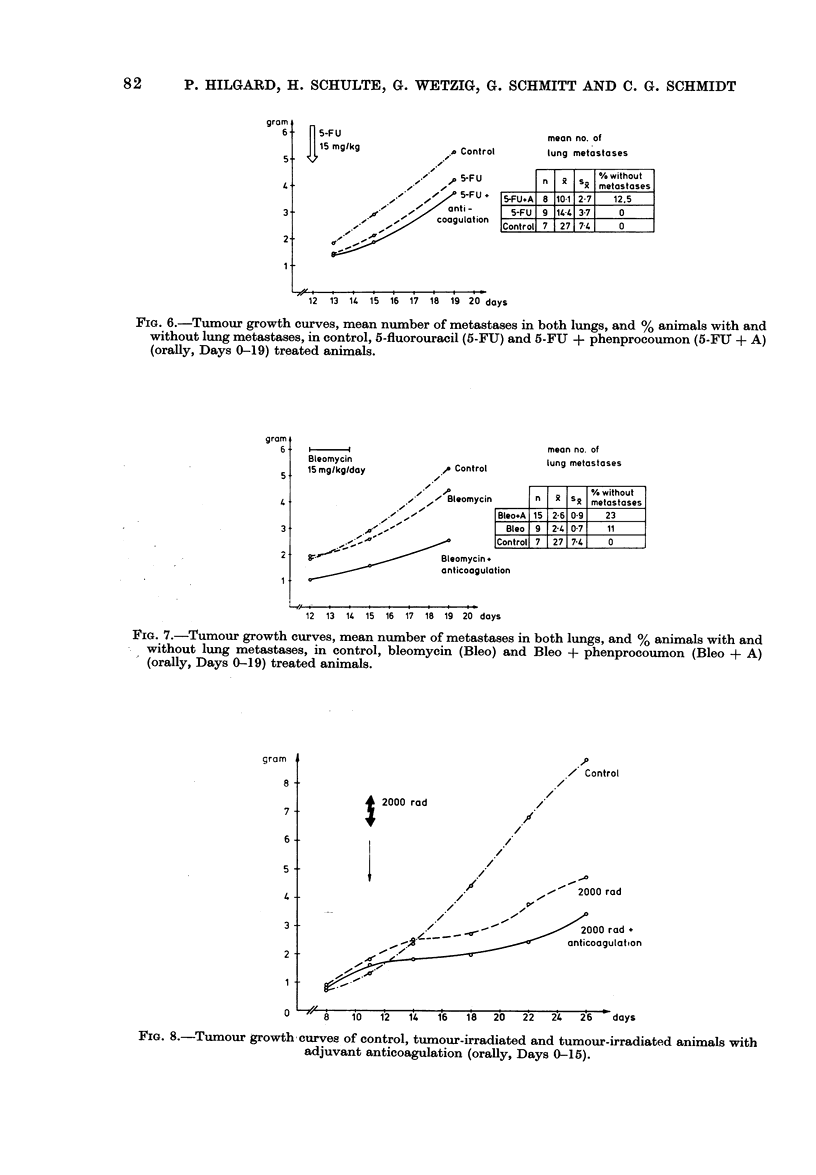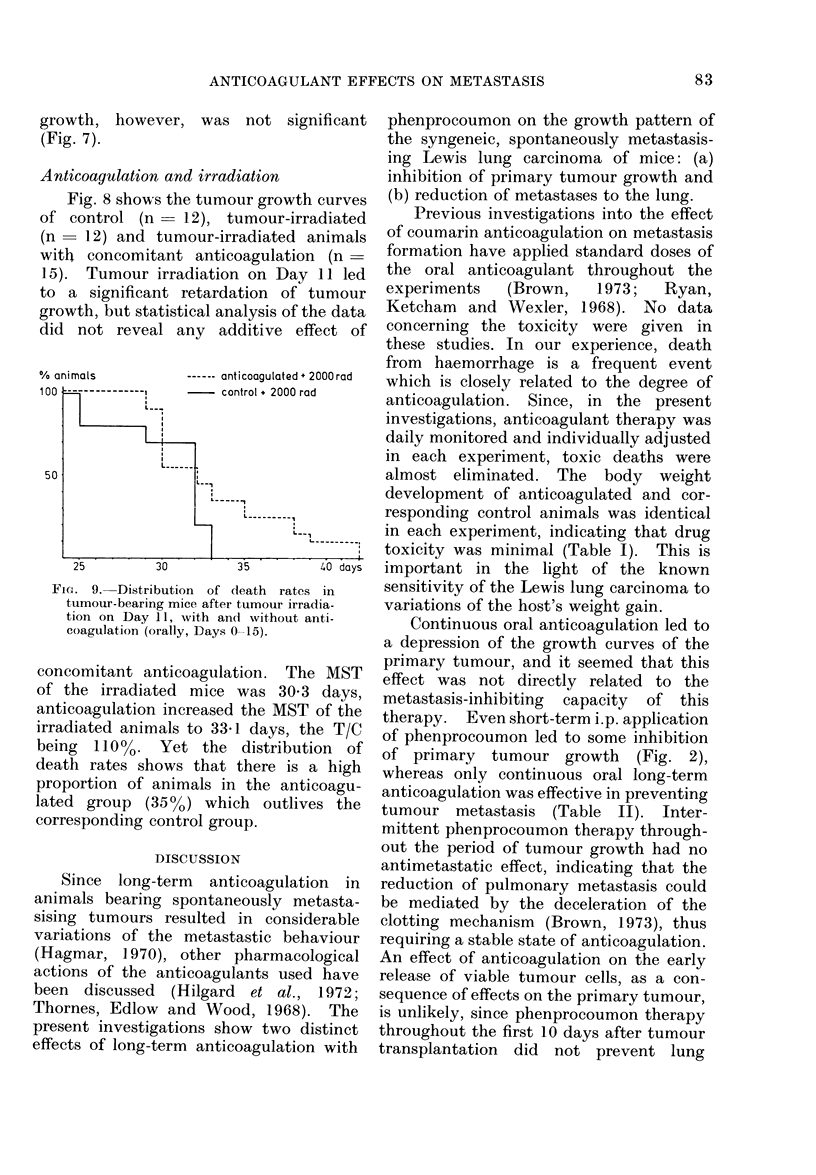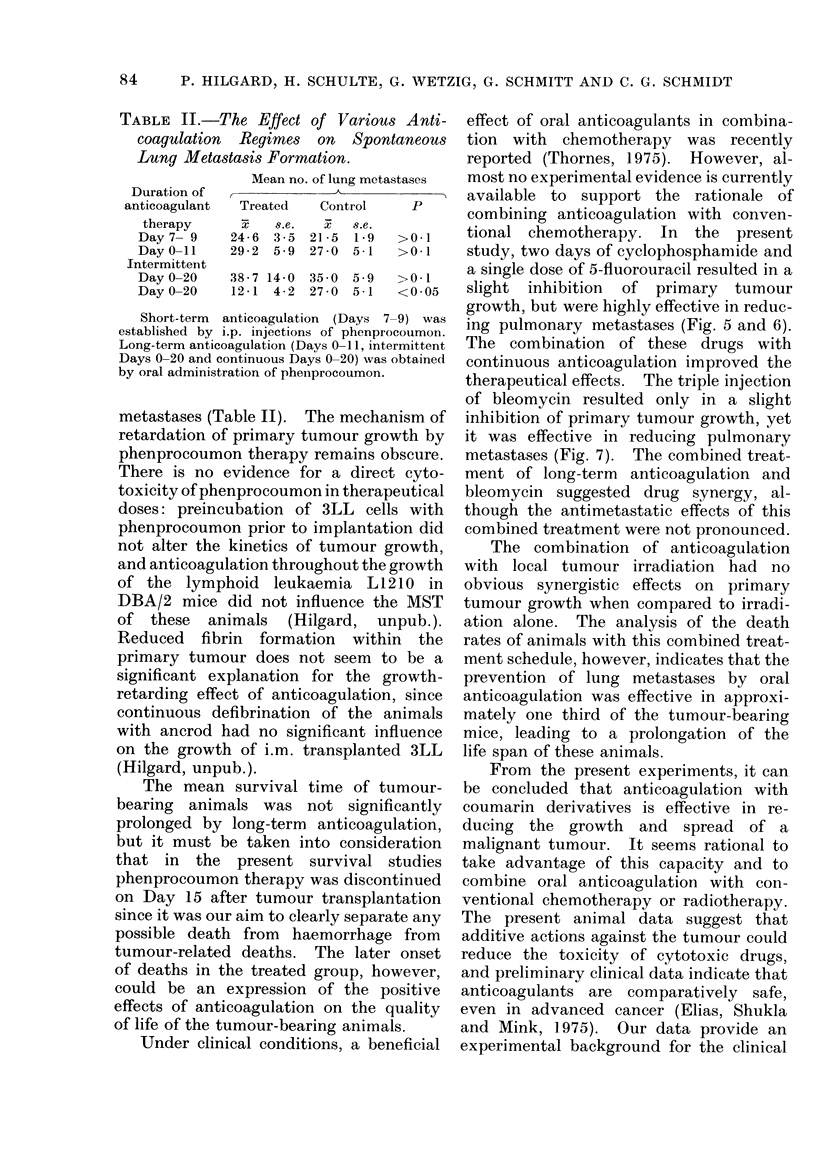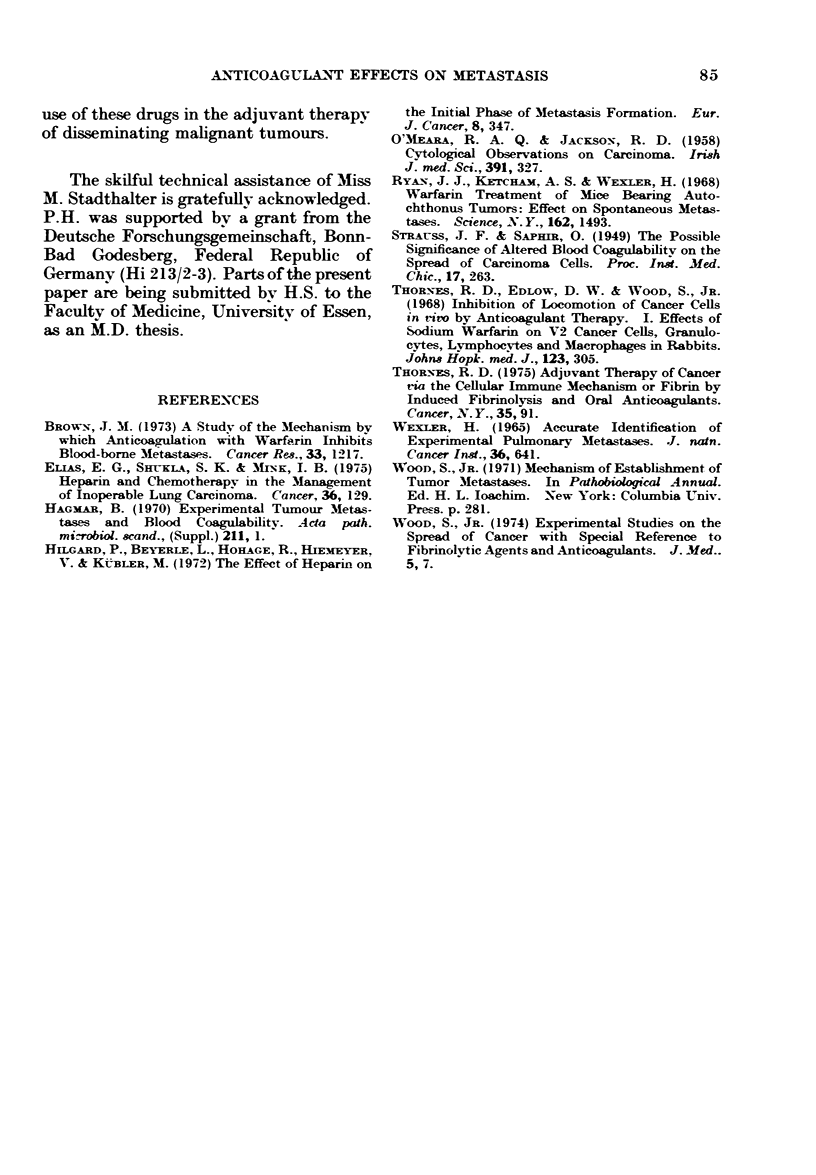# Oral anticoagulation in the treatment of a spontaneously metastasising murine tumour (3LL).

**DOI:** 10.1038/bjc.1977.6

**Published:** 1977-01

**Authors:** P. Hilgard, H. Schulte, G. Wetzig, G. Schmitt, C. G. Schmidt

## Abstract

The effects of long-term anticoagulation with phenprocoumon on growth of the Lewis lung carcinoma (3LL) were studied. Oral anticoagulation initiated at the day of i.m. transplantation of the 3LL into C57BL mice significantly inhibited primary tumour growth and reduced the number of spontaneous metastases to the lungs. Intermittent anticoagulation was without effect on metastasis formation but still retarded primary growth. There was no influence of anticoagulation on the mean survival time (MST) of tumour-bearing animals. Phenprocoumon appears to improve the results of cyclophosphamide of 5-fluorouracil treatment, but there were no statisticially significant differences. In contrast, bleomycin treatment in combination with adjuvant anticoagulation suggested a possible drug synergy. No significant influence of anticoagulation on the response of the primary tumour to irradiattion was found, though the MST of irradiated and anticoagulated animals was greater than in the solely irradiated controls. The present investigations suggest that coumarin derivatives have some direct tumour-inhibiting capacities, but exert their antimetastatic action via deceleration of the blood clotting mechanism.


Br. J. Cancer (1977) 35, 78.

### ORAL ANTICOAGULATION IN THE TREATMENT OF A SPONTANEOUSLY METASTASISING MURINE TUMOUR (3LL)

P. HILGARD, H. SCHULTE, G. WETZIG, G. SCHMITT* AND C. G. SCHMIDT

*From the Innere Universitätsklinik und Poliklinik (Tumorforschung) and *Radiologisches Zentrum, D-43 Essen 1, Hufelandstr. 55, Federal Republic of Germany*

Received 11 June 1976 Accepted 24 August 1976

**Summary.—The effects of long-term anticoagulation with phenprocoumon on growth of the Lewis lung carcinoma (3LL) were studied. Oral anticoagulation initiated at the day of i.m. transplantation of the 3LL into C57BL mice significantly inhibited primary tumour growth and reduced the number of spontaneous metastases to the lungs. Intermittent anticoagulation was without effect on metastasis formation but still retarded primary growth. There was no influence of anticoagulation on the mean survival time (MST) of tumour-bearing animals. Phenprocoumon appears to improve the results of cyclophosphamide or 5-fluorouracil treatment, but there were no statististically significant differences. In contrast, bleomycin treatment in combination with adjuvant anticoagulation suggested a possible drug synergy. No significant influence of anticoagulation on the response of the primary tumour to irradiation was found, though the MST of irradiated and anticoagulated animals was greater than in the solely irradiated controls. The present investigations suggest that coumarin derivatives have some direct tumour-inhibiting capacities, but exert their antimetastatic action via deceleration of the blood clotting mechanism.**

FIBRIN formation around intravascular tumour cell emboli is considered to be of importance for the establishment of haematogenous metastases (Wood, 1971). On the other hand, fibrin has been detected within and around solid tumours, and it was assumed that this would favour the invasive growth of malignant tissue (O'Meara and Jackson, 1958). Under experimental conditions, the pharmacological alteration of the host's clotting mechanism suggested the pathogenic significance of blood coagulation in the growth and dissemination of tumours (Wood, 1974). Various anticoagulants have been used to influence the spread of rodent tumours, yet many of the results reported are contradictory. The experimental systems most widely used were lung colony assays after i.v. injection of tumour cell suspensions. Since these systems are highly artificial and do not correlate with any known clinical condition, the interest has focused on spontaneously metastasising tumours. Since the first report of the effects of dicumarol on circulating Brown–Pearce carcinoma cells in rabbits by Strauss and Saphir (1949), anticoagulation with coumarin derivatives has been a frequent approach to altering the blood coagulability of tumour-bearing animals (Hagmar, 1970).

The present investigations deal with the effects of controlled long-term anticoagulation on primary and metastatic growth of the spontaneously metastasising Lewis lung carcinoma (3LL). Furthermore, some effects of anticoagulation in combination with conventional chemotherapy and radiotherapy were investigated.

### MATERIAL AND METHODS

*Animals.*—C57BL/6 J-Han spf mice of both sexes were used throughout the experiments. The weight range was between 17

and 22 g. The animals were fed with commercial pellets (Altromin ®) and allowed to drink tap water *ad libitum*.

*Lewis lung carcinoma*.—This tumour originated spontaneously as a carcinoma of the lung of a C57BL mouse in Dr Lewis' laboratory at the Wistar Institute in 1951. Our tumour was obtained from Prof. K. Karrer (Institute of Cancer Research, University of Vienna) and it was maintained by s.c. implants of $5 \times 10^6$ tumour cells into C57BL mice, with passage every 14 days. Tumour cell suspensions were obtained by homogenization of solid fragments in sterile saline (containing 250 u/ml streptomycin and 500 u/ml penicillin). The tumour cell count was adjusted to the appropriate concentration with sterile, pyrogen-free saline. Experimental animals were transplanted i.m. with $5 \times 10^6$ tumour cells into the left hind leg.

*Anticoagulation*.—Phenprocoumon (Marcumar ®, Hoffmann–La Roche, Basle) was added to the drinking water at concentrations ranging from 2 mg to 8 mg/l tap water. The degree of anticoagulation was measured by the Thrombotest method: 0·003 ml of tail-vein blood was added to 0·3 ml of Thrombotest reagent (Nyegaard, Oslo) and the clotting time was recorded automatically. Using this method, the normal range in mice (n = 35) was established to be 46–54 s. The phenprocoumon dose was checked daily in order to prolong the Thrombotest clotting time of the experimental animals to 150–200 s throughout the entire experiments. Three different animals from each test group were used in alternating sequence to monitor the clotting times. Unless otherwise stated, anticoagulation was initiated at the days of tumour transplantation and continued until the end of each experiment.

In some experiments, short-term anticoagulation was initiated by i.p. injection of phenprocoumon in a concentration of 2·5 mg/kg body weight every 24 h.

*Chemotherapy*.—(a) Cyclophosphamide (Asta-Werke, Brackwede) was administered i.p. on two consecutive days, in a dose of 30 mg/kg body weight per day, starting Day 7 after tumour transplantation. (b) 5-Fluorouracil (Hoffmann–La Roche, Basle) was given i.p. in a single dose of 15 mg/kg body weight on Day 12 after tumour transplantation. (c) Bleomycin (Mack, Illertissen) was injected on 3 consecutive days i.p. at a dose of 15 mg/kg body weight per day, starting Day 12 after tumour transplantation.

*Irradiation*.—Single doses of 2000 rad were given, using opposing field technique. Ten days after tumour transplantation, the tumour-bearing leg was locally irradiated with a $^{60}Co$ source fitted with a tube collimator of 3 cm diameter. The dose rate was 199 rad/min at an SSD of 46·6 cm. The field inhomogeneity in the target volume was less than $\pm$ 2%.

*Evaluation of tumour growth and lung metastases*.—Tumour growth curves were calculated by the approximate tumour weights from measurement of tumour diameters with a Vernier caliper. The longest and shortest diameters were measured in mm, and the mass expressed in mg by multiplying the length of the tumour by the width squared and dividing the product by 2 (tumour weight (mg) $= l \times w^2/2$). After sacrifice of the animals, the lungs were stained *in situ* through the trachea with 12% Indian ink, and the macroscopically visible lung metastases were counted (Wexler, 1965).

*Survival studies*.—Survival studies were carried out (a) in control and anticoagulated tumour-bearing animals and (b) in irradiated tumour-bearing animals with and without adjuvant anticoagulation. The mean survival time (MST) was calculated after cessation of the anticoagulant therapy on Day 15 after tumour transplantation. The ratio of the MST of the treated group to the MST of the corresponding control group (T/C) was expressed as %.

*Statistical evaluation*.—The statistical analysis of tumour weight and lung metastases data in each experimental group was carried out using the U test for two random variables according to Wilcoxon, Mann and Whitney.

### RESULTS

### *Anticoagulation*

The oral medication of phenprocoumon, with daily adjustment of the dose, resulted in a stable state of anticoagulation throughout the entire study. Death from haemorrhage was infrequent, the loss of animals within a single experiment in the anticoagulated groups never exceeding 10%. During the experiments, all animals were regularly weighed, and the weight gain in control and anticoagulated groups was always identical. An example of the weight development in control (n = 30) and phenprocoumon-treated (n = 40) tumour-bearing animals is given in Table I.

TABLE I.—*Body Weight of Tumour-bearing Animals*

| Days after transplantation | Tumour animal body weight (g) | | | |
|---|---|---|---|---|
| | Control | | Phenprocoumon | |
| | $\bar{x}$ | *s.e.* | $\bar{x}$ | *s.e.* |
| Day 0 | 18·8 | 0·5 | 19·5 | 0·3 |
| Day 12 | 21·2 | 0·3 | 18·6 | 1·0 |
| Day 20 | 22·8 | 0·6 | 22·2 | 0·3 |

Phenprocoumon was added to the drinking water from Days 0–20.

*Effect of anticoagulation on primary tumour growth*

This is shown in Fig. 1 by the growth curves of tumours in control (n = 12) and anticoagulated animals (n = 15). Phenprocoumon was given throughout the entire period of tumour growth. It is obvious that there was a significant inhibition of tumour growth on Day 12 ($P < 0{\cdot}01$). In another experiment, the influence of limited anticoagulation on tumour growth was tested in 3 groups of 10 animals each. Anticoagulation was established by i.p. injections of phenprocoumon. The maximum and most significant effect was seen if the drug was administered from Days 1–10 of tumour growth ($P < 0{\cdot}05$). However, there was still some tumour inhibition by short-term treatment (Days 4–9 and Days 7–9, Fig. 2).

FIG. 1.—Growth curves of the early Lewis lung carcinoma in anticoagulated (orally, Days 0–12) and control mice.

FIG. 2.—Effect of various anticoagulation regimens on tumour weight (g) on Day 12 after transplantation (oral anticoagulation Days 1–10, i.p. anticoagulation Days 4–9 and Days 7–9).

*Metastastic growth of lung tumours*

The metastatic growth of lung tumours in control and anticoagulated animals is shown in Fig. 3. Each group consisted of 30 animals. Oral anticoagulation was maintained from the day of tumour transplantation until the end of the

FIG. 3.—Mean number of metastases in both lungs and % animals with lung metastases in anticoagulated (orally, Days 0–20) and control mice.

experiment (Day 20). The reduction of the mean number of lung metastases in the phenprocoumon-treated group was statistically highly significant ($P = 0{\cdot}002$). Whereas all animals in the control group had lung metastases, only 50% of the anticoagulated animals had macroscopical evidence of lung tumour. As shown in Table II, short-term anticoagulation was ineffective in reducing lung metastases. Only those animals receiving phenprocoumon from Days 0–20 had significantly fewer lung tumours, thus confirming the previous experiment (Fig. 3). Short-term therapy (Days 7–9) was given i.p., whereas treatment from Days 0–11, 0–20 and intermittent anticoagulation (Days 0–20) was oral.

FIG. 4.—Distribution of death rates in tumour-bearing mice with and without anticoagulation (orally, Days 0–15).

*Survival studies*

The MST of 12 control animals bearing the Lewis lung carcinoma was 29·4 days. Anticoagulation from Day 0 to 15 resulted in a MST of 31·1 days in 15 animals, T/C thus being 106%. This difference is not statistically significant. Fig. 4 demonstrates the distribution of the death rates in the two groups. It will be noted that, despite the similar MST, the onset of death of the anticoagulated animals was clearly delayed.

*Anticoagulation and chemotherapy*

In all experiments, phenprocoumon was given from Day 0 until the end of the study (Day 16 or 19). The effect of cyclophosphamide treatment alone, and in combination with anticoagulation, on primary and metastatic tumour growth is shown in Fig. 5. There was a slight flattening of the growth curve after cyclophosphamide treatment; the combination of cyclophosphamide with anticoagulation improved the results somewhat. The reduction in lung metastasis by adjuvant anticoagulation was statistically not significant. Similar effects were found when anticoagulation was combined with 5-fluorouracil treatment (Fig. 6). In contrast, the combination of bleomycin and anticoagulation resulted in a highly significant tumour inhibition when compared to bleomycin alone ($P = 0{\cdot}01$). The effect on the metastatic mean no. of lung metastases

| | n | $\bar{x}$ | $s_{\bar{x}}$ | % without metastases |
|---|---|---|---|---|
| Cyclo+A | 8 | 1·1 | 1·0 | 75 |
| Cyclo | 8 | 0·8 | 0·5 | 50 |
| Control | 10 | 16·8 | 2·9 | 0 |

FIG. 5.—Tumour growth curves, mean number of metastases in both lungs, and % animals with and without lung metastases, in control, cyclophosphamide (Cyclo) and Cyclo + phenprocoumon (Cyclo + A) (orally, Days 0–16) treated animals.

mean no. of lung metastases

| | n | x̄ | $s_{\bar{x}}$ | % without metastases |
|---|---|---|---|---|
| 5-FU+A | 8 | 10·1 | 2·7 | 12,5 |
| 5-FU | 9 | 14·4 | 3·7 | 0 |
| Control | 7 | 27 | 7·4 | 0 |

FIG. 6.—Tumour growth curves, mean number of metastases in both lungs, and % animals with and without lung metastases, in control, 5-fluorouracil (5-FU) and 5-FU + phenprocoumon (5-FU + A) (orally, Days 0–19) treated animals.

mean no. of lung metastases

| | n | x̄ | $s_{\bar{x}}$ | % without metastases |
|---|---|---|---|---|
| Bleo+A | 15 | 2·6 | 0·9 | 23 |
| Bleo | 9 | 2·4 | 0·7 | 11 |
| Control | 7 | 27 | 7·4 | 0 |

FIG. 7.—Tumour growth curves, mean number of metastases in both lungs, and % animals with and without lung metastases, in control, bleomycin (Bleo) and Bleo + phenprocoumon (Bleo + A) (orally, Days 0–19) treated animals.

FIG. 8.—Tumour growth curves of control, tumour-irradiated and tumour-irradiated animals with adjuvant anticoagulation (orally, Days 0–15).

growth, however, was not significant (Fig. 7).

*Anticoagulation and irradiation*

Fig. 8 shows the tumour growth curves of control (n = 12), tumour-irradiated (n = 12) and tumour-irradiated animals with concomitant anticoagulation (n = 15). Tumour irradiation on Day 11 led to a significant retardation of tumour growth, but statistical analysis of the data did not reveal any additive effect of concomitant anticoagulation. The MST of the irradiated mice was 30·3 days, anticoagulation increased the MST of the irradiated animals to 33·1 days, the T/C being 110%. Yet the distribution of death rates shows that there is a high proportion of animals in the anticoagulated group (35%) which outlives the corresponding control group.

FIG. 9.—Distribution of death rates in tumour-bearing mice after tumour irradiation on Day 11, with and without anticoagulation (orally, Days 0–15).

### DISCUSSION

Since long-term anticoagulation in animals bearing spontaneously metastasising tumours resulted in considerable variations of the metastastic behaviour (Hagmar, 1970), other pharmacological actions of the anticoagulants used have been discussed (Hilgard *et al.*, 1972; Thornes, Edlow and Wood, 1968). The present investigations show two distinct effects of long-term anticoagulation with phenprocoumon on the growth pattern of the syngeneic, spontaneously metastasising Lewis lung carcinoma of mice: (a) inhibition of primary tumour growth and (b) reduction of metastases to the lung.

Previous investigations into the effect of coumarin anticoagulation on metastasis formation have applied standard doses of the oral anticoagulant throughout the experiments (Brown, 1973; Ryan, Ketcham and Wexler, 1968). No data concerning the toxicity were given in these studies. In our experience, death from haemorrhage is a frequent event which is closely related to the degree of anticoagulation. Since, in the present investigations, anticoagulant therapy was daily monitored and individually adjusted in each experiment, toxic deaths were almost eliminated. The body weight development of anticoagulated and corresponding control animals was identical in each experiment, indicating that drug toxicity was minimal (Table I). This is important in the light of the known sensitivity of the Lewis lung carcinoma to variations of the host's weight gain.

Continuous oral anticoagulation led to a depression of the growth curves of the primary tumour, and it seemed that this effect was not directly related to the metastasis-inhibiting capacity of this therapy. Even short-term i.p. application of phenprocoumon led to some inhibition of primary tumour growth (Fig. 2), whereas only continuous oral long-term anticoagulation was effective in preventing tumour metastasis (Table II). Intermittent phenprocoumon therapy throughout the period of tumour growth had no antimetastatic effect, indicating that the reduction of pulmonary metastasis could be mediated by the deceleration of the clotting mechanism (Brown, 1973), thus requiring a stable state of anticoagulation. An effect of anticoagulation on the early release of viable tumour cells, as a consequence of effects on the primary tumour, is unlikely, since phenprocoumon therapy throughout the first 10 days after tumour transplantation did not prevent lung

TABLE II.—*The Effect of Various Anticoagulation Regimes on Spontaneous Lung Metastasis Formation.*

| Duration of anticoagulant therapy | Mean no. of lung metastases | | | | |
|---|---|---|---|---|---|
| | Treated | | Control | | $P$ |
| | $\bar{x}$ | *s.e.* | $\bar{x}$ | *s.e.* | |
| Day 7– 9 | 24·6 | 3·5 | 21·5 | 1·9 | $>0\cdot1$ |
| Day 0–11 | 29·2 | 5·9 | 27·0 | 5·1 | $>0\cdot1$ |
| Intermittent Day 0–20 | 38·7 | 14·0 | 35·0 | 5·9 | $>0\cdot1$ |
| Day 0–20 | 12·1 | 4·2 | 27·0 | 5·1 | $<0\cdot05$ |

Short-term anticoagulation (Days 7–9) was established by i.p. injections of phenprocoumon. Long-term anticoagulation (Days 0–11, intermittent Days 0–20 and continuous Days 0–20) was obtained by oral administration of phenprocoumon.

metastases (Table II). The mechanism of retardation of primary tumour growth by phenprocoumon therapy remains obscure. There is no evidence for a direct cytotoxicity of phenprocoumon in therapeutical doses: preincubation of 3LL cells with phenprocoumon prior to implantation did not alter the kinetics of tumour growth, and anticoagulation throughout the growth of the lymphoid leukaemia L1210 in DBA/2 mice did not influence the MST of these animals (Hilgard, unpub.). Reduced fibrin formation within the primary tumour does not seem to be a significant explanation for the growth-retarding effect of anticoagulation, since continuous defibrination of the animals with ancrod had no significant influence on the growth of i.m. transplanted 3LL (Hilgard, unpub.).

The mean survival time of tumour-bearing animals was not significantly prolonged by long-term anticoagulation, but it must be taken into consideration that in the present survival studies phenprocoumon therapy was discontinued on Day 15 after tumour transplantation since it was our aim to clearly separate any possible death from haemorrhage from tumour-related deaths. The later onset of deaths in the treated group, however, could be an expression of the positive effects of anticoagulation on the quality of life of the tumour-bearing animals.

Under clinical conditions, a beneficial effect of oral anticoagulants in combination with chemotherapy was recently reported (Thornes, 1975). However, almost no experimental evidence is currently available to support the rationale of combining anticoagulation with conventional chemotherapy. In the present study, two days of cyclophosphamide and a single dose of 5-fluorouracil resulted in a slight inhibition of primary tumour growth, but were highly effective in reducing pulmonary metastases (Fig. 5 and 6). The combination of these drugs with continuous anticoagulation improved the therapeutical effects. The triple injection of bleomycin resulted only in a slight inhibition of primary tumour growth, yet it was effective in reducing pulmonary metastases (Fig. 7). The combined treatment of long-term anticoagulation and bleomycin suggested drug synergy, although the antimetastatic effects of this combined treatment were not pronounced.

The combination of anticoagulation with local tumour irradiation had no obvious synergistic effects on primary tumour growth when compared to irradiation alone. The analysis of the death rates of animals with this combined treatment schedule, however, indicates that the prevention of lung metastases by oral anticoagulation was effective in approximately one third of the tumour-bearing mice, leading to a prolongation of the life span of these animals.

From the present experiments, it can be concluded that anticoagulation with coumarin derivatives is effective in reducing the growth and spread of a malignant tumour. It seems rational to take advantage of this capacity and to combine oral anticoagulation with conventional chemotherapy or radiotherapy. The present animal data suggest that additive actions against the tumour could reduce the toxicity of cytotoxic drugs, and preliminary clinical data indicate that anticoagulants are comparatively safe, even in advanced cancer (Elias, Shukla and Mink, 1975). Our data provide an experimental background for the clinical use of these drugs in the adjuvant therapy of disseminating malignant tumours.

The skilful technical assistance of Miss M. Stadthalter is gratefully acknowledged. P.H. was supported by a grant from the Deutsche Forschungsgemeinschaft, Bonn-Bad Godesberg, Federal Republic of Germany (Hi 213/2-3). Parts of the present paper are being submitted by H.S. to the Faculty of Medicine, University of Essen, as an M.D. thesis.

### REFERENCES

Brown, J. M. (1973) A Study of the Mechanism by which Anticoagulation with Warfarin Inhibits Blood-borne Metastases. *Cancer Res.*, **33**, 1217.

Elias, E. G., Shukla, S. K. & Mink, I. B. (1975) Heparin and Chemotherapy in the Management of Inoperable Lung Carcinoma. *Cancer*, **36**, 129.

Hagmar, B. (1970) Experimental Tumour Metastases and Blood Coagulability. *Acta path. microbiol. scand.*, (Suppl.) **211**, 1.

Hilgard, P., Beyerle, L., Hohage, R., Hiemeyer, V. & Kübler, M. (1972) The Effect of Heparin on the Initial Phase of Metastasis Formation. *Eur. J. Cancer*, **8**, 347.

O'Meara, R. A. Q. & Jackson, R. D. (1958) Cytological Observations on Carcinoma. *Irish J. med. Sci.*, **391**, 327.

Ryan, J. J., Ketcham, A. S. & Wexler, H. (1968) Warfarin Treatment of Mice Bearing Autochthonus Tumors: Effect on Spontaneous Metastases. *Science, N.Y.*, **162**, 1493.

Strauss, J. F. & Saphir, O. (1949) The Possible Significance of Altered Blood Coagulability on the Spread of Carcinoma Cells. *Proc. Inst. Med. Chic.*, **17**, 263.

Thornes, R. D., Edlow, D. W. & Wood, S., Jr. (1968) Inhibition of Locomotion of Cancer Cells *in vivo* by Anticoagulant Therapy. I. Effects of Sodium Warfarin on V2 Cancer Cells, Granulocytes, Lymphocytes and Macrophages in Rabbits. *Johns Hopk. med. J.*, **123**, 305.

Thornes, R. D. (1975) Adjuvant Therapy of Cancer *via* the Cellular Immune Mechanism or Fibrin by Induced Fibrinolysis and Oral Anticoagulants. *Cancer, N.Y.*, **35**, 91.

Wexler, H. (1965) Accurate Identification of Experimental Pulmonary Metastases. *J. natn. Cancer Inst.*, **36**, 641.

Wood, S., Jr. (1971) Mechanism of Establishment of Tumor Metastases. In *Pathobiological Annual.* Ed. H. L. Ioachim. New York: Columbia Univ. Press. p. 281.

Wood, S., Jr. (1974) Experimental Studies on the Spread of Cancer with Special Reference to Fibrinolytic Agents and Anticoagulants. *J. Med.*, **5**, 7.